# Spatially resolved tissue architecture and computational pathology in pancreatic cancer

**DOI:** 10.1038/s12276-026-01782-4

**Published:** 2026-08-04

**Authors:** Seong-Woo Bae, Aristotelis Tsirigos, Jimin Min, Anirban Maitra

**Affiliations:** 1 Division of Precision Medicine, Department of Medicine, New York University Grossman School of Medicine, New York, NY USA; 2 Applied Bioinformatics Laboratories, New York University Grossman School of Medicine, New York, NY USA; 3 Laura and Isaac Perlmutter Cancer Center, New York University Grossman School of Medicine, NYU Langone Health, New York, NY USA; 4 Department of Medicine, New York University Grossman School of Medicine, New York, NY USA; 5 Department of Pathology, New York University Grossman School of Medicine, New York, NY USA

**Keywords:** Cancer microenvironment, Data integration

## Abstract

Pancreatic ductal adenocarcinoma (PDAC) is a complex disease characterized by high levels of cellular heterogeneity and pronounced microenvironmental remodelling. Dynamic changes during its initiation and progression contribute to resistance to conventional therapies. Building upon key molecular catalogues established by bulk and single-cell profiling studies that have advanced our understanding of PDAC biology, recent advances in spatial biology have provided much-needed insights by elucidating regionally compartmentalized transcriptomic and proteomic programmes within the PDAC microenvironment. In parallel, emerging computational frameworks in digital pathology and artificial intelligence have advanced the field into a high-dimensional, quantitative discipline, particularly for classifying molecular and clinical features from histopathology images. Despite these advancements, integration of these two modalities remains a major challenge. Here, we summarize the convergence of molecular features identified through spatially resolved profiling in PDAC and its precursor lesions, as well as current developments in AI-powered pathology in cancer research. We further propose a multi-modal integration framework that maps molecular states onto morphological and architectural phenotypes, offering a roadmap for spatially informed patient stratification beyond descriptive tissue characterization. We posit that the path forward relies on disciplined cross-scale integration of spatial, histological, and clinical data to ensure meaningful translation into clinical practice.

## Integrating Spatial Biology and Computational Pathology in Pancreatic Cancer

Pancreatic ductal adenocarcinoma (PDAC) is a highly lethal cancer, often diagnosed late and resistant to treatment. This review highlights emerging evidence from spatial profiling studies demonstrating that PDAC and its precursor lesions are not just a simple mixture of malignant and stromal cells but rather a structured ecosystem with spatially distinct immune and fibroblast niches. Digital pathology and artificial intelligence offer promising complementary approaches to extending insights gained from spatial omics to larger patient cohorts, with the potential to improve risk stratification, prognostic prediction and assessment of therapeutic response. Future research should focus on establishing ground-truth characteristics in different disease contexts in the pancreas and expanding spatially resolved datasets across diverse PDAC cohorts to enable robust development of computational frameworks and improve their clinical applications.

## Introduction

Pancreatic cancer remains one of the most lethal malignancies, with a 5-year survival rate of only 13%, caused by late diagnosis, early metastatic spread and profound resistance to conventional therapies^1,2^. Increasing evidence suggests that pancreatic ductal adenocarcinoma (PDAC), the predominant histological subtype in pancreatic cancer, does not always follow a linear trajectory of genetic accumulation during initiation and progression, but can also emerge through epithelial plasticity orchestrated by spatially and temporally constrained microenvironmental cues^3–8^.

Pancreatic stroma is not simply a passive scaffold but an active participant in tumour evolution. The tumour microenvironment (TME) forms spatially distinct niches that can either promote or constrain epithelial phenotypes. In response to dynamic microenvironmental remodelling and cell–cell communication, epithelial cells adopt diverse lineage states and often co-exist under selective pressures imposed by immune, fibroblastic, vascular and metabolic components of the microenvironment. The co-evolution of epithelial and stromal compartments thereby creates permissive or restrictive niches that govern tumour initiation, progression and metastatic potential. Therefore, understanding spatially structured interactions is important for explaining how microenvironmental niches affect tumour behaviour and clinical outcomes.

Over the past decades, substantial efforts have been made to characterize the molecular and cellular heterogeneity of PDAC and its TME. Bulk and single-cell sequencing studies have identified discrete epithelial lineage programmes^9–13^, fibroblast subtypes^14,15^ and immune programmes associated with clinical outcomes^16^. Although these approaches have significantly improved our understanding of PDAC heterogeneity, they provide limited insight into the spatial organization and coordination of cellular states within intact tissue architecture. To address these limitations, emerging spatial profiling technologies, including spatial transcriptomics, spatial proteomics and multiplexed imaging, have begun to bridge this gap by identifying recurrent tissue motifs, cellular neighbourhoods and molecular gradients invisible to the dissociative profiling approaches^17,18^. Utilizing these platforms, it is now possible to interrogate molecular states, cellular identities and tissue architecture in situ, providing new insight into how epithelial plasticity and stromal niches are organized across diverse disease states^19,20^.

Concurrently, whole-slide imaging of routine histopathology has enabled us to facilitate computational approaches to quantifying morphological features, such as glandular architecture, stromal density, immune infiltration patterns, and tumour–stroma interfaces. These approaches also allow large-scale analysis of tissue architecture and spatial heterogeneity across patient cohorts. Recent rapid advances in artificial intelligence (AI), particularly deep learning applied to digitized haematoxylin-and-eosin (H&E) images, have enabled generalizable extraction of meaningful morphological representations without exhaustive manual annotation, achieving high accuracy and performance in cancer detection, histological subtyping and outcome prediction^21–24^. Furthermore, multimodal frameworks that jointly analyse histopathology images, together with clinical and molecular data, are beginning to bridge morphology and functional biology, facilitating clinically actionable insights^25,26^. In PDAC, several approaches have shown early promise in predicting therapy-relevant molecular subtypes directly from morphological features^27,28^. Also, when integrated with spatially resolved omics data, computational frameworks have anchored molecularly defined cell states and tissue niches to their morphological correlates, opening a path towards extending spatial biological insights to larger, clinically relevant patient cohorts^29,30^.

Here, we review current knowledge of PDAC tissue architecture and heterogeneity defined by spatially resolved profiling technologies, summarizing epithelial plasticity and stromal niche dynamics across disease progression, including precursor lesions. We also provide the current role of digital pathology and AI in cancer research and explore how these approaches can provide integrative frameworks to extend spatial biological insights to larger patient populations and improve predictive precision. Finally, we consider the challenges and opportunities in leveraging spatial biology to enhance early detection, risk stratification and clinical translation in this highly refractory disease.

## Main

### Biological insights from spatial omics into tissue architecture and disease states in pancreatic ductal adenocarcinoma and its precursor lesions

#### Spatial organization and heterogeneity in pancreatic ductal adenocarcinoma

Spatial profiling studies have recently redefined PDAC as a highly compartmentalized ecosystem rather than a disordered mixture of malignant cells and stromal cells. Early spatial transcriptomic work, performed prior to the commercial release of the 10x Genomics Visium platform, generated the first region-resolved atlas of PDAC and delineated distinct cancer, immune and fibroblast niches within intact tumour sections^31^. By integrating spatial data with single-cell RNA sequencing, this study demonstrated distinct transcriptomic programmes across ductal epithelium, malignant cells, and stromal compartments that are spatially constrained in a manner consistent with histological architecture. Intriguingly, cancer cells with divergent transcriptomic states preferentially co-localize with specific stromal compartments. For example, inflammatory fibroblasts are often associated with tumour cells enriched for stress–response signatures. Subsequent studies comparing different tumour regions further revealed that the tumour periphery is enriched for hypoxia-associated programmes, glycolytic processes and epithelial–mesenchymal transition signatures compared with tumour centres^32,33^. These peripheral zones are also characterized by increased abundance of myofibroblastic cancer-associated fibroblasts (myCAFs), reinforcing the concept that cancer-cell states and fibroblast subtypes are spatially co-patterned, rather than randomly distributed^32^.

Molecular subtyping efforts based on bulk and single-cell transcriptomics have classified PDAC into classical, basal-like and intermediate/hybrid states with distinct clinical implications^9,10,12,34,35^. Although dissociated single-cell approaches established that multiple tumour lineages can coexist within individual tumours, they could not resolve how these programmes are spatially arranged. Spatially resolved analyses have demonstrated that lineage coexistence maps onto defined architectural contexts and exhibits non-random associations with fibroblast subtypes, particularly in tumour-proximal regions. Laser capture microdissection-based spatial profiling first identified spatially confined stromal states, including “reactive” and “desert” niches defined by distinct fibroblast programmes with divergent immune activity and therapeutic responsiveness^3^. Desert niches appeared to support tumour differentiation and confer chemoprotective properties, whereas reactive niches promoted tumour progression and emergence of basal-like phenotypes. More recent spatial transcriptomic studies have shown that inflammatory CAFs are preferentially enriched in tumour-distal regions, whereas myCAFs localize adjacent to malignant glands^36,37^. In larger cohorts, interspersion of classical, basal-like, and intermediate tumour states has been observed within single tumours, accompanied by structured myCAF enrichment at tumour–stroma interfaces and in basal-like regions^30^. Notably, intermediate tumour states — positioned transcriptionally between classical and basal-like programmes and linked to poor prognosis — exhibit preferential spatial proximity to antigen-presenting CAFs and immunomodulatory niches^30,37^. Whether CAF localization actively drives tumour phenotypic transitions or reflects adaptation to tumour-derived cues remains unresolved. Nonetheless, spatial analyses unequivocally demonstrate that fibroblast heterogeneity is architecturally structured and tightly aligned with malignant cell states.

Beyond tumour–fibroblast coupling, CAF organization has emerged as a central determinant of immune architecture in PDAC. Single-cell analyses previously suggested that fibroblasts can remodel immune landscapes^14,38^, and recent spatial profiling studies have directly visualized immune exclusion within defined tissue niches. Enrichment of myCAFs in tumour-adjacent regions is consistently associated with reduced T cell and plasma-cell infiltration, forming physical and chemokine-mediated barriers at the tumor–stroma interface, and is linked to poor clinical outcomes^30,36,39,40^. These findings position myCAF-rich zones as spatial mediators of immune evasion in PDAC. In addition to the CAF-driven immune exclusion, immune infiltration in PDAC often segregates into immunologically “hot” and “cold” sub-environments. Multiple immunosuppressive niches have been identified and linked to clinical outcomes. There are regions enriched for exhausted CD8^+^ T cells and NKT cells adjacent to immunosuppressive myeloid niches^41^. Regionally localized macrophage populations influence tumour-intrinsic transcriptional dichotomies and contribute to CD8^+^ T cell exclusion, thereby reinforcing spatially confined immunosuppressive ecosystems that can be modulated by immunotherapy and chemotherapy^4^. Finally, close proximity of M2-like macrophages to tumour cells correlates with poor disease-free survival, underscoring the clinical relevance of immune spatial profiling^42^.

Unfortunately, these spatial patterns are not fixed but instead undergo significant remodelling in response to therapy. Comparisons between treatment-naïve and treated tumors have demonstrated that treatment can alter the distribution of surrounding stromal populations, leading to shifts in tumour–CAF interactions and downstream pathways that are rarely observed in untreated specimens^43–46^. In addition, distinct niches enriched for persister-like tumour cells, activated stromal subsets and altered immune infiltration have been identified, suggesting that treatment can also influence localized adaptive responses to therapeutic intervention^47^. These spatially structured changes may contribute to therapeutic resistance by creating a protective microenvironment that can support tumour-cell survival and limit immune-mediated tumour clearance. Together, these findings suggest that therapy drives coordinated changes in cell behaviour, cell–cell interactions and stromal organization within the TME, underscoring the importance of spatial context in understanding treatment response and therapeutic efficacy.

**Fig. 1:**
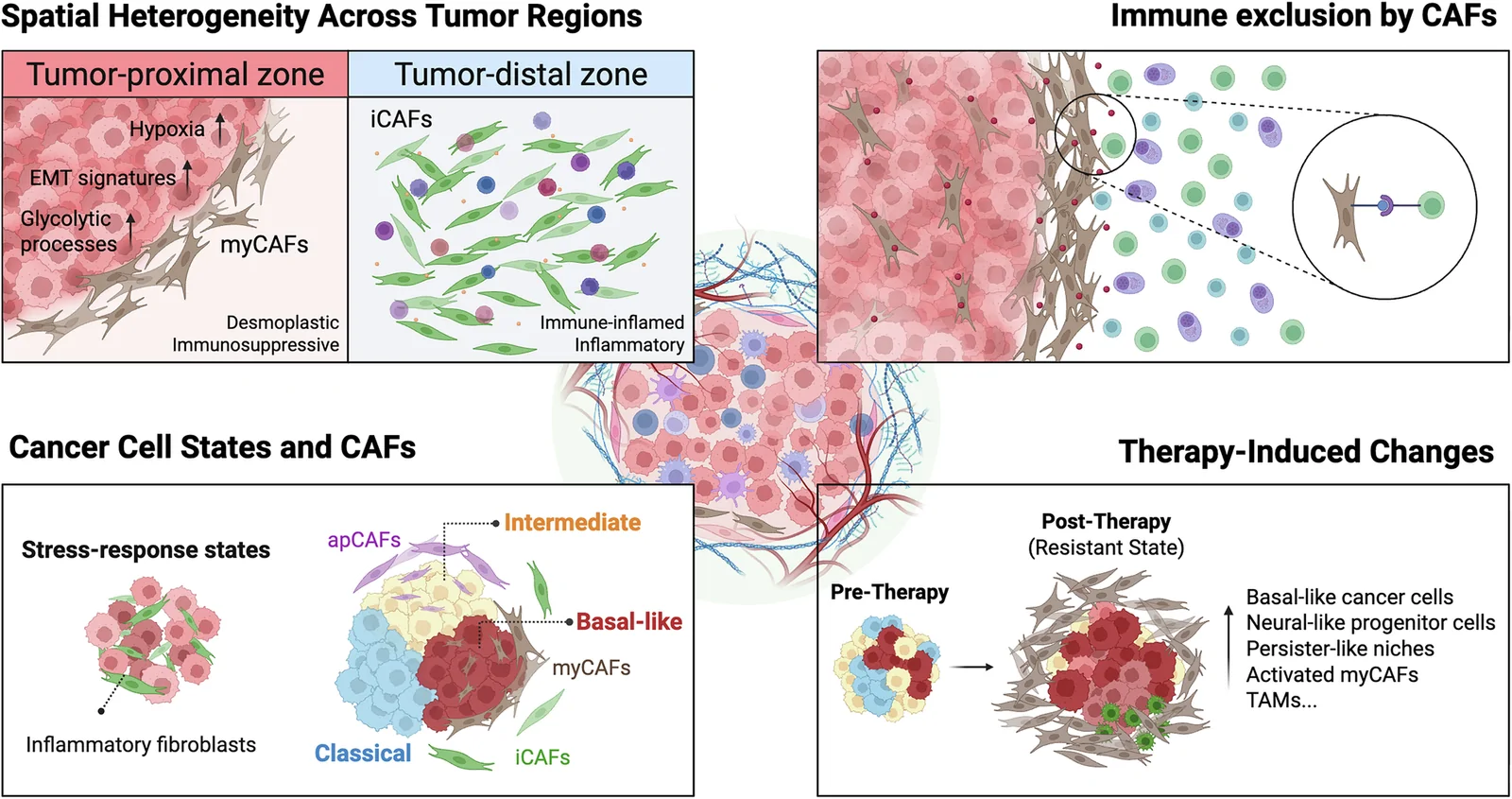
Spatial organization and functional heterogeneity of the pancreatic ductal adenocarcinoma microenvironment. Pancreatic ductal adenocarcinoma (PDAC) stroma exhibits a distinct spatial architecture; tumour-proximal zones are characterized by hypoxia, epithelial-to-mesenchymal transition (EMT) signatures, glycolytic processes and myofibroblastic cancer-associated fibroblast (myCAF) enrichment, whereas tumour-distal zones harbour inflammatory cancer-associated fibroblasts (iCAFs) and immune-inflamed profiles. This heterogeneity facilitates selective crosstalk between specific CAF subtypes, including myCAFs, iCAFs and antigen-presenting cancer-associated fibroblasts (apCAFs), and diverse malignant transcriptional states, such as classical, intermediate and basal-like lineages. CAFs actively drive immune evasion by establishing physical and biochemical barriers that promote immune exclusion from the tumour parenchyma. Following therapeutic intervention, the microenvironment undergoes extensive remodelling towards a resistant state, marked by shifts in cancer-cell plasticity and the expansion of activated myCAFs and tumour-associated macrophages (TAMs).

Collectively, these spatial studies (primarily leveraging transcriptomics) characterize PDAC as a structured disease with evolving tissue ecologies (Fig. 1). Such microenvironmental architectures are dynamically remodelled across tumour regions and under therapeutic pressure, influencing immune exclusion, stromal reprogramming, lineage plasticity and adaptive resistance. To date, most spatial datasets have focused on established tumours, leaving a critical gap in understanding when these architectural rules first emerge. Studying the earliest spatial rewiring events that occur before overt malignancy is necessary for understanding the microenvironmental conditions that permit tumour initiation. In this regard, extending spatial profiling to pancreatic precursor lesions provides an invaluable opportunity to capture the initial steps of epithelial–stromal–immune reorganization that ultimately shape the trajectory towards invasive disease. In the following section, we review current knowledge of pancreatic precursor lesions from a spatial perspective and discuss how early architecture reprogramming may influence PDAC evolution.

#### Early epithelial and microenvironmental remodelling in precursor lesions

**Fig. 2:**
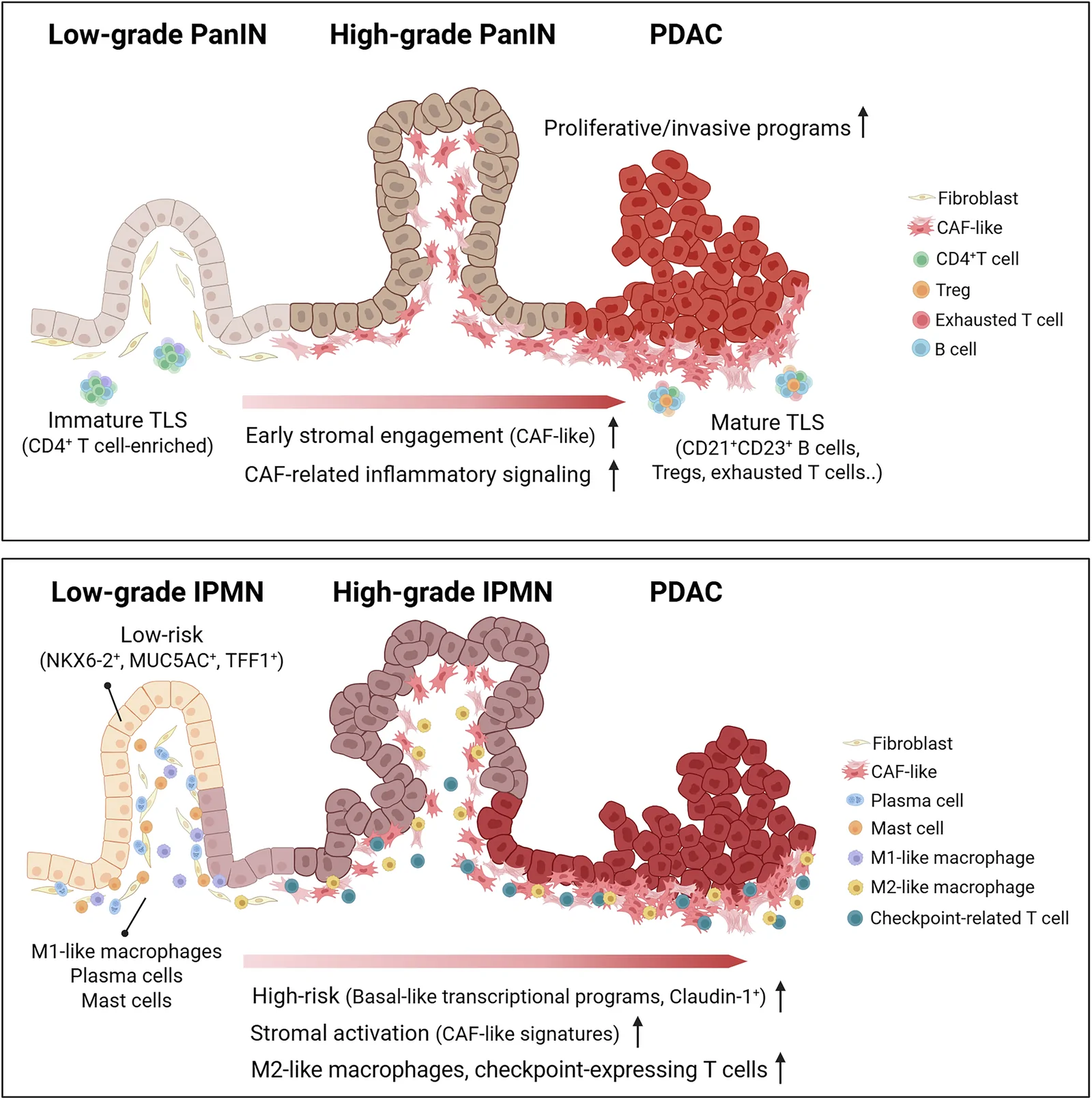
Spatial studies-based molecular landscape during pancreatic ductal adenocarcinoma progression from precursor lesions. The transition to pancreatic ductal adenocarcinoma (PDAC) through distinct precursor pathways — pancreatic intraepithelial neoplasia (PanIN) and intraductal papillary mucinous neoplasm (IPMN) — is marked by progressive stromal activation and immune remodelling. In the PanIN-to-PDAC axis (top), early engagement of cancer-associated fibroblast (CAF)-like cells and inflammatory signalling coincides with changes in tertiary lymphoid structures (TLS), which evolve from immature aggregates to mature structures. Similarly, IPMN progression (bottom) involves a shift from a low-risk state towards a high-risk phenotype, supported by intensified CAF-like signatures, an immunosuppressive shift toward M2-like macrophage polarization and the recruitment of checkpoint-expressing T cells.

Increasing evidence indicates that pancreatic precursor lesions — including pancreatic intraepithelial neoplasia (PanIN) and intraductal papillary mucinous neoplasms (IPMNs) — form spatially differentiate ecosystems in which epithelial plasticity and microenvironmental remodelling develop prior to overt invasion (Fig. 2). Spatially resolved analyses have uncovered molecular divergence across PanIN stages, from low-grade to high-grade lesions^43^. Progression from low-grade to high-grade PanIN is associated with a transition from inflammatory signalling towards proliferative and invasion-related programmes in the epithelium^48^. In addition, fibroblasts surrounding PanIN lesions display CAF-like transcriptional features observed in invasive PDAC, supporting a model in which epithelial–stromal co-evolution begins before malignant transformation^48^. Spatial analyses have also revealed early immune reorganization during the progression. For example, immature tertiary lymphoid structures (TLSs) assemble adjacent to PanIN lesions and are enriched for CD4^+^ T cells, with comparatively lower densities of regulatory T cells and exhausted phenotypes than observed in established PDAC^49^. Together, these findings indicate that PanIN-associated immune architecture is spatially organized yet remains qualitatively distinct from the immune landscapes of invasive disease.

Spatial transcriptomic profiling of IPMN has further refined our understanding of early epithelial diversification. Multiple studies demonstrate coexistence of transcriptionally distinct epithelial programmes within individual lesions, including “low-risk” and “high-risk” states that often parallel classical and basal-like lineages observed in PDAC, even when lesions appear microscopically similar. Notably, high-risk molecular signatures can be detected within histologically low-grade regions^50,51^, underscoring discordance between morphology and molecular risk. Lineage-specifying transcription factors appear to govern these early epithelial trajectories. For example, *NKX6‑2* has been identified as a master regulator of an indolent gastric-like programme, and its loss marks progression towards high-grade dysplasia^52^. Acquisition of basal-like programmes and concomitant loss of exocrine differentiation markers correlate strongly with invasive potential, mirroring lineage transitions in established PDAC^50–52^. Microenvironmental remodelling accompanies these epithelial shifts. High-grade non-invasive IPMNs can already display carcinoma-like epithelial programmes alongside stromal activation and emergence of fibroblasts adopting CAF-like signatures^53,54^. In parallel, immune niches evolve from plasma-cell- and mast-cell-enriched environments in low-grade lesions towards immunosuppressive states marked by M2-like macrophages and checkpoint-associated T cell phenotypes during progression^50,52,54,55^. These findings position IPMN as an evolving spatial ecosystem in which epithelial plasticity and stromal and immune restructuring unfold prior to invasion.

A critical limitation of many precursor-focused studies is their reliance on pancreata harbouring invasive PDAC, raising the possibility of tumour-associated field effects. Establishing a molecular and architectural baseline for the normal exocrine pancreas is therefore essential for distinguishing early oncogenic reprogramming from background tissue variability. Spatial profiling of pancreata from healthy organ donors provides such a reference framework. Multimodel analyses combining multiplex immunohistochemistry, single-cell RNA-seq and GeoMx spatial transcriptomics profiling have revealed that microscopic PanIN lesions are surprisingly common in ostensibly healthy organs across a wide age range^56^. Although donor-derived sporadic PanINs share core transcriptional features with those from PDAC-bearing pancreata, tumour-associated lesions exhibit enhanced basal-like gene expression and evidence of early molecular divergence. Moreover, the surrounding stroma in donor pancreata differs substantially from peritumoral PDAC stroma, with distinct fibroblast and macrophage signatures, suggesting that elements of microenvironmental activation can arise in isolation but remain spatially constrained in the absence of invasive disease^56^. Complementary spatial proteomic analyses further indicate that molecular reprogramming may precede overt histological alteration. PanINs arising within tumour-bearing pancreata exhibit a pronounced field effect characterized by activation of stress adaptation, immune engagement and metabolic rewiring programmes, whereas PanINs from healthy donors largely retain a quiescent molecular state^57^. These data define early spatially patterned molecular states that may prime tissue regions for subsequent invasion.

Not only do organ-donor-derived spatial maps provide a reference for sporadic preneoplastic lesions but they also enable rigorous benchmarking against normal pancreatic tissue architecture. As summarized in Table 1, this area is growing, with multiple donor-derived spatial datasets emerging across diverse platforms, including GeoMx, CosMx, Visium, Xenium and spatially resolved proteomics. Although comprehensive mapping of the normal human pancreas remains in its early stages and available resources are still limited in number, these studies already capture regional anatomical heterogeneity and microenvironmental variation across different cell compartments or states. Importantly, they also document disease-relevant perturbations, such as diabetes or cystic fibrosis, thereby establishing an essential baseline against which cancer-associated spatial programmes can be interpreted. Integrating organ-donor atlases with datasets from precursor lesions and invasive PDAC will be critical for disentangling early pathogenic reprogramming from normal tissue variability and for reconstructing the initial spatial trajectories that culminate in pancreatic tumorigenesis.

**Table 1:** Publicly available spatial omics datasets generated from human organ donor pancreas.

Cohort	Sample type	Dataset	Refs.
Healthy organ donor pancreas with incidental PanINs	FFPE (1 donor; 2 sections)	GeoMx Digital Spatial Profiling whole-transcriptome profiling	^56^
Anatomically resolved normal pancreas atlas across head/body/tail regions	FFPE (16 donors; 64 sections)	Xenium Prime 5 K (+ custom probes), CODEX proteomic imaging	^95^
Normal glucose tolerance vs type 2 diabetes donors	Frozen (6 donors; 16 sections)	Visium whole-transcriptome (non-CytAssist)	^96^
Non-diabetic vs type 1 diabetes donors	FFPE (6 donors)	CosMx Spatial Molecular Imaging (~ 1,000-plex + custom genes)	^97^
Cystic fibrosis (CF)/CF-related diabetes (CFRD) vs control donors	FFPE (12 donors)	CosMx (~ 1,000-plex)	^98^
Non-diabetic vs type 1 diabetes cohort	FFPE (20 donors)	Visium CytAssist whole-transcriptome	^99^
Incidental PanIN lesions in cancer-free donor pancreas	FFPE (10 donors; 11 sections)	Deep Visual Proteomics	^57^

FFPE, formalin-fixed paraffin-embedded.

In summary, spatial profiling studies across PDAC and its precursor lesions show that cancer development and progression are closely associated with progressive remodelling of tissue architecture, driven by dynamic changes among diverse cell populations. These datasets outline a spatial continuum that links normal pancreatic structure to early neoplastic alterations and, ultimately, to the complex tumour ecosystems in invasive PDAC. Although current spatial omics technologies provide fundamental biological insights, their broader application remains limited by resources, technical requirements, and challenges in standardization across multi-cohort studies and platforms. Thus, most investigations are still restricted to relatively small sample sizes and specialized sample preparation. There is a critical need for approaches that can translate detailed spatial atlases into clinically useful interpretations applicable to larger and more diverse patient populations. Digital pathology and AI-driven image analysis have emerged as practical and highly complementary strategies. Using routine histological slides, computational models trained on whole-slide images (WSIs) establish scalable and quantitative assessment of tissue architecture and spatial patterning across large clinical samples. These approaches can capture morphological features correlated with epithelial states, stromal structure and immune distribution, thereby connecting molecularly defined spatial atlases with large-scale studies of pancreatic cancer progression. Integrating AI-driven histomorphology analysis with spatial omics frameworks represents a promising research direction for translating spatial biology into clinically scalable diagnostics and prognostic tools. In the sections below, we discuss several compelling developments in digital pathology and AI in this field and outline potential strategies for their integration with spatial biology approaches.

### Digital pathology and computational analysis of pancreatic ductal adenocarcinoma histology

Digital pathology converts conventional histology slides into computationally accessible WSIs, typically from H&E-stained tissue sections, now routinely acquired at gigapixel scale^58^. Conventional histopathological assessment, while remaining the clinical gold standard, is constrained by inter-observer variability and limited scalability for capturing the complex spatial features characteristic of PDAC, including the heterogeneous admixture of tumour glands, desmoplastic stroma and variable immune infiltrates^59–61^. Digital pathology addresses these limitations through standardized visualization, large-scale data sharing and computational analysis of tissue morphology, and has become the primary substrate for AI-based methods. Quantitative characterization of tumour architecture and microenvironmental composition across whole-tissue sections can capture features that are difficult to assess by manual review alone. WSIs with gigapixel-scale size present substantial computational challenges and are therefore typically processed using patch-based representations at predefined magnifications^62,63^. This enables scalable computation while preserving fine-grained histological detail.

Workflows based on patch extraction can facilitate multi-scale analysis by enabling models to integrate information across spatial resolutions, ranging from local cellular and nuclear patterns captured at higher magnifications to tissue-level architecture at lower magnifications^62^. Multi-scale representations are well suited to PDAC, where tumour–stroma boundaries, immune infiltration density, and glandular architecture can differ substantially between adjacent tissue regions. Furthermore, pipelines commonly include tissue detection/segmentation, artefact filtering, and stain or colour normalization (or augmentation) to mitigate technical variability from staining and scanning, which can improve robustness and generalizability across cohorts and institutions^64^.

The section below describes AI methodologies that operate on these images, and their specific applications to PDAC.

#### Supervised learning approaches

In early stages of AI applications in digital pathology, supervised learning with annotated WSIs was utilized for tasks such as tumour detection, histological grading and classification. Landmark studies in detection of breast-cancer metastasis^65^, Gleason grading of prostate cancer^66^ and classification of lung-cancer subtype^67^ evaluated that deep-learning models can match or exceed pathologist-level performance when trained on large annotated datasets. Supervised approaches in PDAC have been applied to tumour detection in endoscopic ultrasound-guided fine-needle biopsy specimens, where low tumour cellularity and tissue fragmentation pose diagnostic challenges^68^. Deep-learning-based segmentation of tumour epithelium and stromal compartments has further enabled quantitative characterization of the PDAC TME from routine H&E slides, including automated estimation of tumor–stroma ratios with prognostic relevance^69^ and identification of stromal and lymphocyte features associated with survival^70^. However, supervised learning is constrained by the need for extensive expert annotations for training. In PDAC, this is a bottleneck, where region-level labelling of extreme histological heterogeneity marked by pathologists is extensively laborious and potential inter-observer disagreement exists.

#### Weakly supervised and multiple-instance learning

The reliance of supervised learning on detailed spatial annotations has motivated weakly supervised approaches that leverage slide-level labels^63^. Multiple-instance learning (MIL) treats a WSI as a “bag” of image patches and trains models using slide-level labels such as diagnosis, survival or molecular status, without requiring explicit annotation of informative regions. This is well suited to histopathology, where slide-level clinical and molecular annotations are often available at scale, whereas pixel- or region-level delineations are labour intensive. MIL-based approaches have demonstrated clinically relevant predictions across cancer types, including survival prediction in mesothelioma without region-level annotations^71^ and attention-based frameworks that both classify slides and localize diagnostically informative regions^62^. Weakly supervised models have also shown that mutation status, gene-expression patterns and transcriptomic subtypes can be inferred from H&E WSIs without matched, detailed spatial annotations^72^, a practical advantage for scaling to large retrospective cohorts.

In PDAC, Saillard et al. applied a weakly supervised framework to predict transcriptomic subtypes and stratify prognosis from routine H&E slides^28^. As large annotated PDAC image datasets remain scarce, slide-level learning strategies are particularly attractive for this disease. Notably, this approach revealed image-derived features not captured by standard pathology review, including tile-level subtype maps that expose intratumoural heterogeneity, minor aggressive subtype components and spatial tumor–stroma mixing patterns that are associated with prognosis. These findings illustrate that weakly supervised models can not only classify PDAC tumours but also resolve morphological heterogeneity at a spatial resolution finer than conventional assessment.

#### Representation learning and foundation models

Recent work has shifted towards representation learning that can learn generalizable feature embeddings from histopathology images that transfer across downstream tasks^22,73^. In practice, pretrained representations are commonly used as fixed-feature extractors with lightweight downstream models, as initialization for task-specific fine-tuning, or for embedding-based retrieval and clustering^22,74^. Patch-level embeddings typically require aggregation for whole-slide applications, often implemented using attention-based pooling or MIL approaches, to produce slide-level representations^62,73^. Rather than training task-specific features from scratch, these models reuse pretrained visual embeddings adaptable across tissue types and disease contexts. Building on these advances, the alignment of histology embeddings and spatial omics embeddings is opening new possibilities for predicting spatial molecular states and cell-type composition directly from H&E images, a direction with promise for mapping the complex PDAC microenvironment from routine slides at scale^75^. More recently, multimodal vision-language models have aligned histology image embeddings with pathology text, including report-derived supervision, to support retrieval, report generation, and other cross-modal applications^23,76^.

A paramount concern of pathology foundation models is to improve robustness in domain shift arising from differences in staining, tissue processing and scanning protocols across institutions^21,77^. For PDAC, where multi-institutional cohorts are essential but tissue processing and staining vary considerably across centres, foundation-model representations may help to mitigate the domain shift that has limited the generalizability of earlier models.

#### Prognostic, predictive and biomarker applications in pancreatic ductal adenocarcinoma

A major translational objective is to derive prognostic and treatment-relevant predictions from routine histology. In PDAC, weakly supervised whole-slide models have stratified patients by survival from H&E images alone^28,78^. As noted in the supervised approach section above, a deep-learning model based on stromal and immune features extracted from histology have been associated with survival^70^, suggesting that both learned representations and interpretable morphological characteristics carry prognostic information.

Beyond prognosis, earlier studies have linked image-derived signatures to treatment outcomes in clinically treated cohorts. An AI-derived histological signature was associated with disease-specific survival among patients receiving adjuvant gemcitabine^79^, and separate efforts have reported prediction of postoperative recurrence from digitized histology in resected PDAC cohorts that received adjuvant therapy^80^. These treatment-associated biomarkers represent a class of image-derived markers that complement molecular profiling. A notable direction is histology-to-transcriptomics inference, linking local morphology to molecular profiles. Ahmadvand et al. explored H&E-based molecular subtyping in PDAC using subtype labels calculated by gene expressions, motivating prospective evaluation in clinical settings^27^. These signatures in PDAC provide a natural interface with spatial profiling technologies, enabling cross-modal validation and spatially resolved interpretation of morphological heterogeneity.

### Integration with spatial biology

Spatial transcriptomic and proteomic technologies provide a molecular reference for validating AI-derived morphological signals from routine histology. Spatial profiling can anchor and contextualize image model readouts, whereas histology can extend spatial insights as a scalable companion modality to larger cohorts where spatial profiling remains impractical^18,81^.

**Fig. 3:**
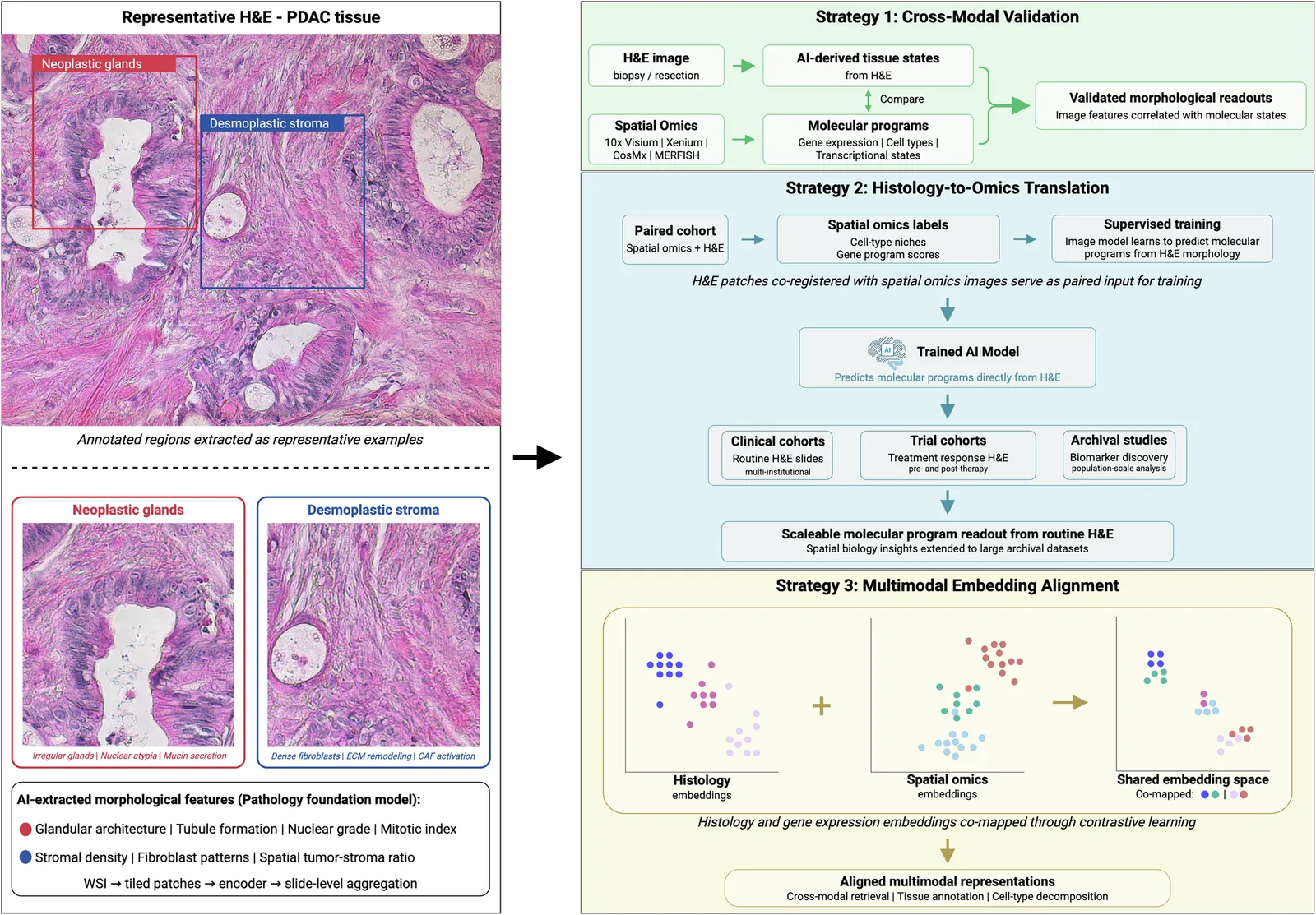
Integration of spatial omics with artificial intelligence-based histology analysis in pancreatic ductal adenocarcinoma. A representative haematoxylin and eosin (H&E) section from a pancreatic ductal adenocarcinoma (PDAC) resection specimen illustrates the two dominant tissue compartments used as morphological inputs: neoplastic glands (red) and desmoplastic stroma (blue). In Strategy 1 (cross-modal validation), image-derived tissue states are compared against molecular programmes measured by spatial omics, anchoring AI predictions to a molecular reference. In Strategy 2 (histology-to-omics translation), spatial omics measurements from a paired cohort provide supervised labels to train an image model that predicts molecular programmes from routine H&E at scale. In Strategy 3 (multimodal embedding alignment), histology-patch embeddings from foundation models and gene-expression embeddings from spatial profiling are mapped into a shared representation space through contrastive learning, enabling direct assessment of morphology–transcriptome correspondence and supporting downstream applications such as cross-modal retrieval, tissue annotation and cell-type decomposition.

We consider three strategies for integrating spatial omics with histology-based image analysis (Fig. 3). The first integration strategy is cross-modal validation, in which image-derived tissue states or spatial patterns inferred from H&E images are compared against spatially resolved molecular programmes measured either on matched tissue sections or across independently profiled cohorts. Second, histology-to-omics translation is one of possible scenarios, where spatial omics labels or spatially defined niches serve as supervision to train image models that predict molecular programmes from H&E images at scale. Recent tools have advanced this direction through histology-guided high-resolution spatial transcriptomics^82,83^ and large-scale biomarker discovery from pathology archives^84^. Continued development of benchmarking resources such as paired spatial transcriptomics and WSI datasets will be important for maturing this integration framework^85^. The third strategy, which has emerged more recently, is multimodal embedding alignment. Histology and gene-expression embeddings from co-registered tissue sections are projected into a shared representation space through contrastive learning, rather than comparing predictions or transferring labels across modalities^75^. The resulting aligned embeddings can then be used for tasks such as cross-modal retrieval, tissue annotation from bulk expression references, or cell-type decomposition guided by both image and transcriptomic features.

In PDAC, these integration strategies are particularly relevant given the spatially confined nature of tumour-stromal niches. As noted above, spatial transcriptomic analyses of treatment-naive and therapy-exposed PDAC have revealed multicellular dynamics and treatment-induced remodelling of tumour and stromal compartments, including spatially restricted driver programmes and transitional cell populations that cooperate with specific microenvironmental states^43–45^. These spatially resolved treatment-response or resistance phenotypes are one of the natural targets for cross-modal validation with AI-based histological models, as treatment-associated morphological changes are often visible on H&E but lack standardized quantitative descriptors. A practical workflow for PDAC could therefore use spatial omics to discover and validate spatially localized biology in smaller deeply profiled cohorts, then deploy AI-enabled pathology to apply these findings as quantitative readouts across larger multi-institutional cohorts. The pronounced desmoplastic stroma of PDAC, whose glandular and stromal patterns on H&E encode distinct microenvironmental states, may be particularly amenable to such histology-to-spatial-programme prediction, although this remains to be systematically tested.

### Current limitations and practical considerations

Although the field of AI for digital pathology has moved quickly, several practical bottlenecks continue to limit the reliable application of AI to PDAC histology and its translation into clinical use. One primary obstacle is inter-institutional generalization. Differences in tissue processing, fixation and staining protocols, scanner hardware, image resolution and patient demographics introduce substantial domain shift, often degrading model performance. Pathology foundation models have been proposed as ways to address this^86^, but none of these is guaranteed to hold up when applied to new datasets. For example, training cohorts cannot fully capture subtle morphological variance, particularly for cancers with tremendous heterogeneity, such as PDAC. Also, these generalization challenges extend beyond algorithmic refinement. Deploying models in a clinical setting requires standardized quality assurance, multi-institutional validation and alignment with regulatory and accreditation standards. Current guidance has accordingly shifted emphasis from standalone performance metrics towards end-to-end workflow validation^87–89^.

Pathology foundation models have improved feature extraction and cross-task transferability, but PDAC remains underrepresented in most pretraining datasets^22,73^. It remains untested whether their embeddings adequately capture the morphological distinctions that matter in this disease, such as classical versus basal-like glandular patterns, activated versus quiescent stroma, or the spatial mixing of tumour and stromal compartments. This gap is compounded at the slide level, where predictions still rely on clinical labels such as survival or recurrence that conflate tumour biology with adjuvant therapy regimens, surgical margin status and patient-level variables^90,91^. Cohort curation, confounder modelling and transparent reporting of cohort composition are still needed to make sure that predictions reflect actual biology rather than artefacts of study design. On the interpretability side, attention-based heatmaps are commonly used for patch attribution but can end up reflecting shortcut learning rather than biologically meaningful signals. Counterfactual analysis and structured stress testing offer more rigorous ways to check whether model outputs actually correspond to meaningful tissue patterns such as tumour–stroma interfaces or stromal activation^92,93^.

Finally, data governance and reproducibility are practical barriers that should not be underestimated. The computational cost of WSI analysis, combined with privacy constraints and institutional reluctance around data sharing, makes cross-institutional benchmarking difficult in practice. Moving forward, standardized evaluation protocols, prospective endpoint preregistration and transparent documentation of dataset provenance and domain characteristics will all be needed. The scarcity of paired histology–spatial transcriptomics datasets for PDAC in existing benchmarks^94^ is another bottleneck, as it limits the ability to perform cross-modal validation of image-derived predictions.

## Concluding remarks

Spatial omics profiling has shown that cellular programmes and microenvironmental states in PDAC and its precursor lesions are often regionally restricted rather than uniformly distributed. These insights emphasize that underlying tumour biology of PDAC requires a spatially informed view, in which epithelial cells, stromal components and immune signatures are considered in relation to their local tissue context.

Along with the spatial omics technologies, digital pathology and AI-based image analysis offer a complementary and scalable perspective on tissue organization. Although spatial omics provide high-resolution molecular insight in deeply characterized samples, histology-based computational modelling allows us to assess tissue architecture and microenvironmental patterns across larger patient cohorts. Together, these approaches support a multiscale view that links morphology, cellular states and molecular programmes.

In PDAC, the integration of spatial biology with computational pathology is beginning to refine how tumour heterogeneity, subtype organization and microenvironmental structure are interpreted. Image-derived representations of tissue architecture, subtype mixtures and stromal or immune patterns may function as digital surrogates for underlying biologic states, whereas spatial profiling provides essential context for understanding what these image-based signals represent. This reciprocal relationship allows spatial findings to inform image-based models and unveiled histological features by AI to extend spatially derived concepts to clinically diverse cohorts.

Prospectively, progress is likely to depend less on any single technology than on the coherent integration of modalities. Spatial platforms may define where specific cellular and molecular programs occur, digital pathology may quantify how frequently and in what configurations these patterns appear across patients and clinical data may clarify which features are most relevant to prognosis and therapy. Such cross-scale integration could yield more biologically grounded stratification and improve the interpretability of both molecular and image-derived biomarkers in PDAC.

## References

[1] Siegel, R. L., Kratzer, T. B., Wagle, N. S., Sung, H. & Jemal, A. Cancer statistics, 2026. *CA Cancer J Clin* **76**, e70043. 10.3322/caac.70043 (2026). 41528114 PMC12798275

[2] Stoop, T. F. et al. Pancreatic cancer. *Lancet* **405**, 1182–1202 (2025). 40187844 10.1016/S0140-6736(25)00261-2

[3] Grunwald, B. T. et al. Spatially confined sub-tumor microenvironments in pancreatic cancer. *Cell* **184**, 5577–5592.e18 (2021). 34644529 10.1016/j.cell.2021.09.022

[4] Klein, L. et al. Spatial tumor immune heterogeneity facilitates subtype co-existence and therapy response in pancreatic cancer. *Nat. Commun.* **16**, 335 (2025). 39762215 10.1038/s41467-024-55330-7 PMC11704331

[5] Ligorio, M. et al. Stromal microenvironment shapes the intratumoral architecture of pancreatic cancer. *Cell* **178**, 160–175.e27 (2019). 31155233 10.1016/j.cell.2019.05.012 PMC6697165

[6] Makohon-Moore, A. & Iacobuzio-Donahue, C. A. Pancreatic cancer biology and genetics from an evolutionary perspective. *Nat. Rev. Cancer* **16**, 553–565 (2016). 27444064 10.1038/nrc.2016.66 PMC5739515

[7] Notta, F. et al. A renewed model of pancreatic cancer evolution based on genomic rearrangement patterns. *Nature* **538**, 378–382 (2016). 27732578 10.1038/nature19823 PMC5446075

[8] Yachida, S. et al. Distant metastasis occurs late during the genetic evolution of pancreatic cancer. *Nature* **467**, 1114–1117 (2010). 20981102 10.1038/nature09515 PMC3148940

[9] Bailey, P. et al. Genomic analyses identify molecular subtypes of pancreatic cancer. *Nature* **531**, 47–52 (2016). 26909576 10.1038/nature16965

[10] Collisson, E. A. et al. Subtypes of pancreatic ductal adenocarcinoma and their differing responses to therapy. *Nat. Med.* **17**, 500–503 (2011). 21460848 10.1038/nm.2344 PMC3755490

[11] Dreyer, S. B. et al. Genomic and molecular analyses identify molecular subtypes of pancreatic cancer recurrence. *Gastroenterology* **162**, 320–324.e4 (2022). 34534536 10.1053/j.gastro.2021.09.022 PMC8721486

[12] Moffitt, R. A. et al. Virtual microdissection identifies distinct tumor- and stroma-specific subtypes of pancreatic ductal adenocarcinoma. *Nat. Genet.* **47**, 1168–1178 (2015). 26343385 10.1038/ng.3398 PMC4912058

[13] Raghavan, S. et al. Microenvironment drives cell state, plasticity, and drug response in pancreatic cancer. *Cell* **184**, 6119–6137.e26 (2021). 34890551 10.1016/j.cell.2021.11.017 PMC8822455

[14] Elyada, E. et al. Cross-species single-cell analysis of pancreatic ductal adenocarcinoma reveals antigen-presenting cancer-associated fibroblasts. *Cancer Discov.* **9**, 1102–1123 (2019). 31197017 10.1158/2159-8290.CD-19-0094 PMC6727976

[15] Ohlund, D. et al. Distinct populations of inflammatory fibroblasts and myofibroblasts in pancreatic cancer. *J. Exp. Med.* **214**, 579–596 (2017). 28232471 10.1084/jem.20162024 PMC5339682

[16] Steele, N. G. et al. Multimodal mapping of the tumor and peripheral blood immune landscape in human pancreatic cancer. *Nat. Cancer* **1**, 1097–1112 (2020). 34296197 10.1038/s43018-020-00121-4 PMC8294470

[17] Lewis, S. M. et al. Spatial omics and multiplexed imaging to explore cancer biology. *Nat. Methods* **18**, 997–1012 (2021). 34341583 10.1038/s41592-021-01203-6

[18] Liu, Y., Dai, Y. & Wang, L. Spatial omics at the forefront: emerging technologies, analytical innovations, and clinical applications. *Cancer Cell* **44**, 24–49 (2026). 41478277 10.1016/j.ccell.2025.12.009 PMC12867001

[19] Li, Y. et al. Spatial transcriptomics in pancreatic cancer: advances, prospects and challenges. *Crit. Rev. Oncol. Hematol.* **203**, 104430. 10.1016/j.critrevonc.2024.104430 (2024). 38942220

[20] Kim, J. S. Spatial transcriptomics in pancreatic cancer: current insights and future directions. *J. Dig. Cancer Res.* **13**, 288–301 (2025).

[21] Campanella, G. et al. A clinical benchmark of public self-supervised pathology foundation models. *Nat. Commun.* **16**, 3640 (2025). 40240324 10.1038/s41467-025-58796-1 PMC12003829

[22] Chen, R. J. et al. Towards a general-purpose foundation model for computational pathology. *Nat. Med.* **30**, 850–862 (2024). 38504018 10.1038/s41591-024-02857-3 PMC11403354

[23] Ding, T. et al. A multimodal whole-slide foundation model for pathology. *Nat. Med.* **31**, 3749–3761 (2025). 41193692 10.1038/s41591-025-03982-3 PMC12618242

[24] Wang, X. et al. A pathology foundation model for cancer diagnosis and prognosis prediction. *Nature* **634**, 970–978 (2024). 39232164 10.1038/s41586-024-07894-z PMC12186853

[25] Lu, M. Y. et al. A visual-language foundation model for computational pathology. *Nat. Med.* **30**, 863–874 (2024). 38504017 10.1038/s41591-024-02856-4 PMC11384335

[26] Xiang, J. et al. A vision-language foundation model for precision oncology. *Nature* **638**, 769–778 (2025). 39779851 10.1038/s41586-024-08378-w PMC12295649

[27] Ahmadvand, P. et al. A deep learning approach for the identification of the molecular subtypes of pancreatic ductal adenocarcinoma based on whole slide pathology images. *Am. J. Pathol.* **194**, 2302–2312 (2024). 39222907 10.1016/j.ajpath.2024.08.006 PMC12179518

[28] Saillard, C. et al. Pacpaint: a histology-based deep learning model uncovers the extensive intratumor molecular heterogeneity of pancreatic adenocarcinoma. *Nat. Commun.* **14**, 3459 (2023). 37311751 10.1038/s41467-023-39026-y PMC10264377

[29] Chen, R. J. et al. Pan-cancer integrative histology-genomic analysis via multimodal deep learning. *Cancer Cell* **40**, 865–878.e6 (2022). 35944502 10.1016/j.ccell.2022.07.004 PMC10397370

[30] Pei, G. et al. Spatial mapping of transcriptomic plasticity in metastatic pancreatic cancer. *Nature* **642**, 212–221 (2025). 40269162 10.1038/s41586-025-08927-x PMC13242257

[31] Moncada, R. et al. Integrating microarray-based spatial transcriptomics and single-cell RNA-seq reveals tissue architecture in pancreatic ductal adenocarcinomas. *Nat. Biotechnol.* **38**, 333–342 (2020). 31932730 10.1038/s41587-019-0392-8

[32] Alver, T. N. et al. Spatial transcriptomics reveals cancer and stromal cell heterogeneity between center and invasive front of pancreatic cancer. *Mod. Pathol.* **38**, 100726. 10.1016/j.modpat.2025.100726 (2025). 39889965

[33] Sun, H. et al. Hypoxic microenvironment induced spatial transcriptome changes in pancreatic cancer. *Cancer Biol. Med.* **18**, 616–630 (2021). 34086429 10.20892/j.issn.2095-3941.2021.0158 PMC8185871

[34] Chan-Seng-Yue, M. et al. Transcription phenotypes of pancreatic cancer are driven by genomic events during tumor evolution. *Nat. Genet.* **52**, 231–240 (2020). 31932696 10.1038/s41588-019-0566-9

[35] Puleo, F. et al. Stratification of pancreatic ductal adenocarcinomas based on tumor and microenvironment features. *Gastroenterology* **155**, 1999–2013.e3 (2018). 30165049 10.1053/j.gastro.2018.08.033

[36] Croft, W. et al. Spatial determination and prognostic impact of the fibroblast transcriptome in pancreatic ductal adenocarcinoma. *Elife* 10.7554/eLife.86125 (2023). 37350578 PMC10361717

[37] Kim, S. et al. Integrative analysis of spatial and single-cell transcriptome data from human pancreatic cancer reveals an intermediate cancer cell population associated with poor prognosis. *Genome Med.* **16**, 20 (2024). 38297291 10.1186/s13073-024-01287-7 PMC10832111

[38] Lakins, M. A., Ghorani, E., Munir, H., Martins, C. P. & Shields, J. D. Cancer-associated fibroblasts induce antigen-specific deletion of CD8^+^ T cells to protect tumour cells. *Nat. Commun.* **9**, 948 (2018). 29507342 10.1038/s41467-018-03347-0 PMC5838096

[39] Carstens, J. L. et al. Spatial computation of intratumoral T cells correlates with survival of patients with pancreatic cancer. *Nat. Commun.* **8**, 15095. 10.1038/ncomms15095 (2017). 28447602 PMC5414182

[40] Liu, Y. et al. Conserved spatial subtypes and cellular neighborhoods of cancer-associated fibroblasts revealed by single-cell spatial multi-omics. *Cancer Cell* **43**, 905–924.e6 (2025). 40154487 10.1016/j.ccell.2025.03.004 PMC12074878

[41] Yousuf, S. et al. Spatially resolved multi-omics single-cell analyses inform mechanisms of immune dysfunction in pancreatic cancer. *Gastroenterology* **165**, 891–908.e14 (2023). 37263303 10.1053/j.gastro.2023.05.036

[42] Vayrynen, S. A. et al. Composition, spatial characteristics, and prognostic significance of myeloid cell infiltration in pancreatic cancer. *Clin. Cancer Res.* **27**, 1069–1081 (2021). 33262135 10.1158/1078-0432.CCR-20-3141 PMC8345232

[43] Cui Zhou, D. et al. Spatially restricted drivers and transitional cell populations cooperate with the microenvironment in untreated and chemo-resistant pancreatic cancer. *Nat. Genet.* **54**, 1390–1405 (2022). 35995947 10.1038/s41588-022-01157-1 PMC9470535

[44] Hwang, W. L. et al. Single-nucleus and spatial transcriptome profiling of pancreatic cancer identifies multicellular dynamics associated with neoadjuvant treatment. *Nat. Genet.* **54**, 1178–1191 (2022). 35902743 10.1038/s41588-022-01134-8 PMC10290535

[45] Shiau, C. et al. Spatially resolved analysis of pancreatic cancer identifies therapy-associated remodeling of the tumor microenvironment. *Nat. Genet.* **56**, 2466–2478 (2024). 39227743 10.1038/s41588-024-01890-9 PMC11816915

[46] Werba, G. et al. Single-cell RNA sequencing reveals the effects of chemotherapy on human pancreatic adenocarcinoma and its tumor microenvironment. *Nat. Commun.* **14**, 797 (2023). 36781852 10.1038/s41467-023-36296-4 PMC9925748

[47] Bernard, V. et al. (2025). A spatial atlas of chemoradiation therapy in pancreatic cancer identifies cellular and microenvironmental determinants of persister populations. bioRxiv. 10.1101/2025.06.20.660757.

[48] Bell, A. T. F. et al. PanIN and CAF transitions in pancreatic carcinogenesis revealed with spatial data integration. *Cell Syst.* **15**, 753–769.e5 (2024). 39116880 10.1016/j.cels.2024.07.001 PMC11409191

[49] Lyman, M. R. et al. Spatial proteomics and transcriptomics reveal early immune cell organization in pancreatic intraepithelial neoplasia. *JCI Insight*10.1172/jci.insight.191595 (2025). 40569674 PMC12333942

[50] Iyer, M. K. et al. Spatial transcriptomics of intraductal papillary mucinous neoplasms reveals divergent indolent and malignant states. *Clin. Cancer Res.* **31**, 1796–1808 (2025). 39969959 10.1158/1078-0432.CCR-24-1529 PMC12045729

[51] Li, J. et al. Spatial analysis of IPMNs defines a paradoxical KRT17-positive, low-grade epithelial population harboring malignant features. *Cell Mol. Gastroenterol. Hepatol.* 10.1016/j.jcmgh.2026.101749 (2026). 41638478 PMC13000727

[52] Sans, M. et al. Spatial transcriptomics of intraductal papillary mucinous neoplasms of the pancreas identifies NKX6-2 as a driver of gastric differentiation and indolent biological potential. *Cancer Discov.* **13**, 1844–1861 (2023). 37285225 10.1158/2159-8290.CD-22-1200 PMC10880589

[53] Iyer, M. K. et al. Digital spatial profiling of intraductal papillary mucinous neoplasms: Toward a molecular framework for risk stratification. *Sci. Adv.* **9**, eade4582. 10.1126/sciadv.ade4582 (2023). 36930707 PMC10022906

[54] Peng, S. et al. Evolution of the spatial transcriptomic landscape during the progression of high-grade pancreatic intraductal papillary mucinous neoplasms to invasive cancer. *Pancreatology*10.1016/j.pan.2025.12.012 (2025). 41455615

[55] Cui, M. et al. Spatial transcriptomics defines the molecular progression, invasion and immune landscape of IPMN and IPMN-derived pancreatic cancer. *Gut*10.1136/gutjnl-2025-336117 (2025). 41381181

[56] Carpenter, E. S. et al. Analysis of donor pancreata defines the transcriptomic signature and microenvironment of early neoplastic lesions. *Cancer Discov.* **13**, 1324–1345 (2023). 37021392 10.1158/2159-8290.CD-23-0013 PMC10236159

[57] Min, J. et al. AI-powered deep visual proteomics reveals critical molecular transitions in pancreatic cancer precursors. *Cancer Discov.* **16**, 1323-1340 (2026). 10.1158/2159-8290.CD-25-1119 PMC13289816 42013410

[58] Farahani, N., Parwani, A. & Pantanowitz, L. Whole slide imaging in pathology: advantages, limitations, and emerging perspectives. *Pathol. Lab. Med. Int.* **7**, 23–33 (2015).

[59] Paech, D. C. et al. A systematic review of the interobserver variability for histology in the differentiation between squamous and nonsquamous non-small cell lung cancer. *J. Thorac. Oncol.* **6**, 55–63 (2011). 21107286 10.1097/JTO.0b013e3181fc0878

[60] Rutgers, J. J. et al. Interobserver variability between experienced and inexperienced observers in the histopathological analysis of Wilms tumors: a pilot study for future algorithmic approach. *Diagn. Pathol.* **16**, 77 (2021). 34419100 10.1186/s13000-021-01136-w PMC8380406

[61] Makhlouf, H. R., Ossandon, M. R., Farahani, K., Lubensky, I. & Harris, L. N. Digital pathology imaging artificial intelligence in cancer research and clinical trials: an NCI workshop report. *J. Pathol. Inform.* **20**, 100531. 10.1016/j.jpi.2025.100531 (2026). 41476571 PMC12753273

[62] Lu, M. Y. et al. Data-efficient and weakly supervised computational pathology on whole-slide images. *Nat. Biomed. Eng.* **5**, 555–570 (2021). 33649564 10.1038/s41551-020-00682-w PMC8711640

[63] Campanella, G. et al. Clinical-grade computational pathology using weakly supervised deep learning on whole slide images. *Nat. Med.* **25**, 1301–1309 (2019). 31308507 10.1038/s41591-019-0508-1 PMC7418463

[64] Tellez, D. et al. Quantifying the effects of data augmentation and stain color normalization in convolutional neural networks for computational pathology. *Med. Image Anal.* **58**, 101544. 10.1016/j.media.2019.101544 (2019). 31466046

[65] Ehteshami Bejnordi, B. et al. Diagnostic assessment of deep learning algorithms for detection of lymph node metastases in women with breast cancer. *JAMA* **318**, 2199–2210 (2017). 29234806 10.1001/jama.2017.14585 PMC5820737

[66] Nagpal, K. et al. Development and validation of a deep learning algorithm for Gleason grading of prostate cancer from biopsy specimens. *JAMA Oncol.* **6**, 1372–1380 (2020). 32701148 10.1001/jamaoncol.2020.2485 PMC7378872

[67] Coudray, N. et al. Classification and mutation prediction from non-small cell lung cancer histopathology images using deep learning. *Nat. Med.* **24**, 1559–1567 (2018). 30224757 10.1038/s41591-018-0177-5 PMC9847512

[68] Naito, Y. et al. A deep learning model to detect pancreatic ductal adenocarcinoma on endoscopic ultrasound-guided fine-needle biopsy. *Sci. Rep.* **11**, 8454 (2021). 33875703 10.1038/s41598-021-87748-0 PMC8055968

[69] Vendittelli, P. et al. Automatic quantification of tumor-stroma ratio as a prognostic marker for pancreatic cancer. *PLoS ONE* **19**, e0301969 (2024). 38771787 10.1371/journal.pone.0301969 PMC11108171

[70] Tan, X. et al. Stroma and lymphocytes identified by deep learning are independent predictors for survival in pancreatic cancer. *Sci. Rep.* **15**, 9415 (2025). 40108402 10.1038/s41598-025-94362-x PMC11923104

[71] Courtiol, P. et al. Deep learning-based classification of mesothelioma improves prediction of patient outcome. *Nat. Med.* **25**, 1519–1525 (2019). 31591589 10.1038/s41591-019-0583-3

[72] Schmauch, B. et al. A deep learning model to predict RNA-Seq expression of tumours from whole slide images. *Nat. Commun.* **11**, 3877 (2020). 32747659 10.1038/s41467-020-17678-4 PMC7400514

[73] Xu, H. et al. A whole-slide foundation model for digital pathology from real-world data. *Nature* **630**, 181–188 (2024). 38778098 10.1038/s41586-024-07441-w PMC11153137

[74] Chen, C. et al. Fast and scalable search of whole-slide images via self-supervised deep learning. *Nat. Biomed. Eng.* **6**, 1420–1434 (2022). 36217022 10.1038/s41551-022-00929-8 PMC9792371

[75] Chen, W. et al. A visual-omics foundation model to bridge histopathology with spatial transcriptomics. *Nat. Methods* **22**, 1568–1582 (2025). 10.1038/s41592-025-02707-1 PMC12240810 40442373

[76] Lu, M. Y. et al. A multimodal generative AI copilot for human pathology. *Nature* **634**, 466–473 (2024). 38866050 10.1038/s41586-024-07618-3 PMC11464372

[77] Stacke, K., Eilertsen, G., Unger, J. & Lundstrom, C. Measuring domain shift for deep learning in histopathology. *IEEE J. Biomed. Health Inform.* **25**, 325–336 (2021). 33085623 10.1109/JBHI.2020.3032060

[78] Liu, W. et al. A novel deep learning-based pathomics score for prognostic stratification in pancreatic ductal adenocarcinoma. *Pancreas* **54**, e430–e441 (2025). 40314741 10.1097/MPA.0000000000002463

[79] Nimgaonkar, V. et al. Development of an artificial intelligence-derived histologic signature associated with adjuvant gemcitabine treatment outcomes in pancreatic cancer. *Cell Rep. Med.* **4**, 101013. 10.1016/j.xcrm.2023.101013 (2023). 37044094 PMC10140610

[80] Yamaguchi, R. et al. Machine learning of histopathological images predicts recurrences of resected pancreatic ductal adenocarcinoma with adjuvant treatment. *Pancreas* **53**, e199–e204 (2024). 38127849 10.1097/MPA.0000000000002289

[81] Gong, D., Arbesfeld-Qiu, J. M., Perrault, E., Bae, J. W. & Hwang, W. L. Spatial oncology: translating contextual biology to the clinic. *Cancer Cell* **42**, 1653–1675 (2024). 39366372 10.1016/j.ccell.2024.09.001 PMC12051486

[82] Fu, X., and Chen, Y. (2024). Pix2Path: Integrating Spatial Transcriptomics and Digital Pathology with Deep Learning to Score Pathological Risk and Link Gene Expression to Disease Mechanisms. bioRxiv, 2024.2008.2018.608468. 10.1101/2024.08.18.608468.

[83] Zhang, D. et al. Inferring super-resolution tissue architecture by integrating spatial transcriptomics with histology. *Nat. Biotechnol.* **42**, 1372–1377 (2024). 38168986 10.1038/s41587-023-02019-9 PMC11260191

[84] Shulman, E. D. et al. AI-predicted spatial transcriptomics unlocks breast cancer biomarkers from pathology. *Cell* (2026). 10.1016/j.cell.2026.04.023 PMC13317733 42105763

[85] Glettig, M., Ehrensperger, T., Yates, J., and Boeva, V. (2025). H&Enium, Applying Foundation Models to Computational Pathology and Spatial Transcriptomics to Learn an Aligned Latent Space. bioRxiv, 2025.2007.2022.665986. 10.1101/2025.07.22.665986.

[86] Asadi-Aghbolaghi, M. et al. Learning generalizable AI models for multi-center histopathology image classification. *NPJ Precis. Oncol.* **8**, 151 (2024). 39030380 10.1038/s41698-024-00652-4 PMC11271637

[87] Sebastian, M. et al. Applications and challenges of utilizing digital pathology and AI-enabled workflows in clinical trials. *J. Pathol. Inform.* **20**, 100542. 10.1016/j.jpi.2025.100542 (2026). 41631127 PMC12861029

[88] Szylberg, L. et al. Guidelines for the adoption of digital pathology in clinical pathology units recommended by the polish society of pathologists. *Diagn. Pathol.* **21**, 13 (2026). 41618426 10.1186/s13000-026-01762-2 PMC12874716

[89] Zhang, D. Y. et al. Implementation of digital pathology and artificial intelligence in routine pathology practice. *Lab. Invest.* **104**, 102111. 10.1016/j.labinv.2024.102111 (2024). 39053633

[90] Hanna, M. G. et al. Future of artificial intelligence-machine learning trends in pathology and medicine. *Mod. Pathol.* **38**, 100705. 10.1016/j.modpat.2025.100705 (2025). 39761872

[91] Komura, D., Ochi, M. & Ishikawa, S. Machine learning methods for histopathological image analysis: updates in 2024. *Comput. Struct. Biotechnol. J.* **27**, 383–400 (2025). 39897057 10.1016/j.csbj.2024.12.033 PMC11786909

[92] Kaczmarzyk, J. R., Saltz, J. H., and Koo, P. K. (2024). Explainable AI for computational pathology identifies model limitations and tissue biomarkers. ArXiv.

[93] Klauschen, F. et al. Toward explainable artificial intelligence for precision pathology. *Annu. Rev. Pathol.* **19**, 541–570 (2024). 37871132 10.1146/annurev-pathmechdis-051222-113147

[94] Jaume, G. et al. HEST-1k: a dataset for spatial transcriptomics and histology image analysis. In *Proc. jaume 2024 hest*, *Advances in Neural Information Processing Systems* (2024).

[95] Mereu, E. et al. (2025). Latent plasticity of the human pancreas across development, health, and disease. bioRxiv. 10.1101/2025.10.01.679230.

[96] Howell, N. et al. A spatial transcriptomics dataset of pancreas sections in normal glucose tolerance and type 2 diabetic donors. *Sci. Data* **12**, 1526 (2025). 40890166 10.1038/s41597-025-05450-6 PMC12402493

[97] Melton, R. et al. Single-cell multiome and spatial profiling reveals pancreas cell type-specific gene regulatory programs of type 1 diabetes progression. *Sci. Adv.* **11**, eady0080. 10.1126/sciadv.ady0080 (2025). 40929272 PMC12422192

[98] Mummey, H. M. et al. (2025). Single cell and spatial characterization of the human pancreas reveals drivers of beta cell dysfunction in cystic fibrosis. bioRxiv. 10.64898/2025.12.14.694120.

[99] Medina-Serpas, M. A. et al. Spatial transcriptomics from pancreas and local draining lymph node tissue reveals a lymphotoxin-beta signature in human type 1 diabetes. *Cell Rep.* **45**, 117144 (2026). 10.1016/j.celrep.2026.117144 PMC13202731 41875135

